# Construction of micro-nano robots: living cells and functionalized biological cell membranes

**DOI:** 10.3389/fbioe.2023.1277964

**Published:** 2023-09-12

**Authors:** Jiawen Niu, Chenlu Liu, Xiaopeng Yang, Wenlong Liang, Yufu Wang

**Affiliations:** ^1^ Department of Orthopedic Surgery, The Second Affiliated Hospital of Harbin Medical University, Harbin, China; ^2^ Department of Breast Surgery, The Second Affiliated Hospital of Harbin Medical University, Harbin, China

**Keywords:** micro-nano robots, micromotors, living cells and cell membranes, drug delivery, immunotherapy, phototherapy

## Abstract

Micro-nano robots have emerged as a promising research field with vast potential applications in biomedicine. The motor is the key component of micro-nano robot research, and the design of the motor is crucial. Among the most commonly used motors are those derived from living cells such as bacteria with flagella, sperm, and algal cells. Additionally, scientists have developed numerous self-adaptive biomimetic motors with biological functions, primarily cell membrane functionalized micromotors. This novel type of motor exhibits remarkable performance in complex media. This paper provides a comprehensive review of the structure and performance of micro-nano robots that utilize living cells and functionalized biological cell membranes. We also discuss potential practical applications of these mirco-nano robots as well as potential challenges that may arise in future development.

## 1 Introduction

The field of robotics has experienced a rapid development, resulting in their widespread use in various industries. These robots come in different sizes, ranging from microscopic to macroscopic, with the latter being commonly used in industrial production, service industries, and military warfare. However, the size of macroscopic robots limits their application in certain situations, such as *in vivo* interventional diagnosis and treatment (Soto et al., 2022). This limitation has led to the emergence of micro-nano robots (Wang and Pumera, 2015; Esteban-Fernández et al., 2018; Venugopalan et al., 2020; Wang and Zhang, 2021; Wang X et al., 2022), which are capable of operating on the micro-nano scale and exhibit remarkable flexibility and adaptability. Furthermore, it is capable of working collaboratively within a cluster (Law et al., 2022). In recent decades, micro-nano robots have emerged as a promising field of research with applications in biomedicine, particularly in targeted drug delivery and disease diagnosis and treatment (Zhang et al., 2022a; Wang et al., 2023; Zhang L et al., 2023).

Over the past decade, significant advancements have been made in the design and production of micromotors that utilize various driving methods (Hines et al., 2017; Wang C et al., 2018; Venugopalan et al., 2020). These artificial micro-nano robots can be powered by different propulsion mechanisms including magnetic actuation (Huang et al., 2022; T et al., 2018; Yu et al., 2022), light-actuated (Kadiri et al., 2021), chemical propulsion that relies on surrouding chemical fuels (Xu et al., 2020), ultrasound propulsion (Wu et al., 2014) as well as biohybrid propulsion (Carlsen and Sitti, 2014; Ceylan et al., 2017; Buss et al., 2020; Zhang et al., 2022b; Zhang B et al., 2023). These methods allow micro-nano robots to operate in complex fluid environments and perform biomedically relevant tasks such as targeted drug delivery (Singh et al., 2019; Ji et al., 2021) and precision surgery (Nguyen et al., 2018). This progress has positioned artificial micromotors as a leading area of biomedical research. While there have been successful reports of animal experiments (Gao et al., 2015; Oral and Pumera, 2023) there are still several critical issues that must be addressed before micro-nano robots can be safely and effectively applied to living systems and translated into practical applications. The reliability and biosafety of micromotors depend on synthetic materials, which can trigger immune reactions and contamination in complex living systems. Thus, for *in vivo* application of micromotors, it is crucial to design them biomimetically and ensure their biocompatibility to ensure their efficient operation in physiological environments without any adverse effects.

The combination of micromotor capabilities with the biological properties of native cells have led to the development of living cell-based micromotors (Li et al., 2017; Venugopalan et al., 2020). This innovative approach offers new possibilities to overcome current obstacles faced by micromotors in biomedical operations. By drawing inspiration from nature, the basic biological functions of natural cells, including immune immunity, antigen presentation, cell or tissue-specific targeting, and selective binding of bacteria or pathogens can be harnessed and synthesized into motile micro-nanorobots. But living cells sometimes can fully meet the needs of micromotor’s construction. Cell membrane coating technology is an advanced and powerful technology that utilizes complex proteins, channels, and biological functions of target cell membranes (Li T. et al., 2018; Fang et al., 2018). The coupling of cell membrane and micromotor produces new biocompatible devices that offer a wide range of possibilities for various applications in the field of biomedicine, generating great attention and interest among researchers (Gao et al., 2016; Le et al., 2021).

**FIGURE 1:**
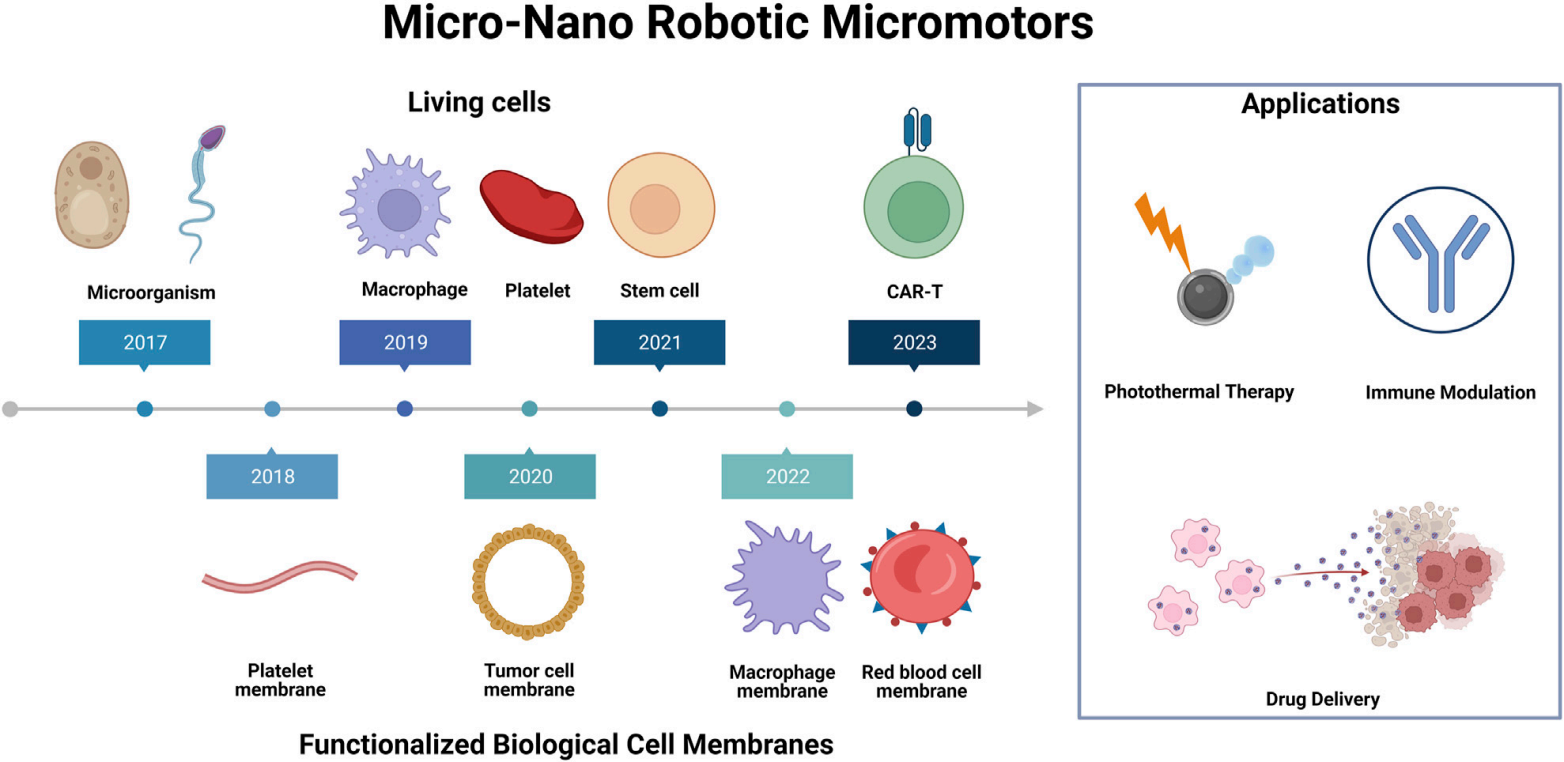
Living cells and functionalized biological cell membranes micro-nano robots for biomedical applications. Illustration presents a schematic representation of various synthetic micro-nano robotic micromotors, which are functionalized by different cells and cell membranes for a variety of biomedical applications, including photothermal therapy, immune modulation and drug delivery.

This review delves into the latest research on the synthesis of micromotors using living cells and functionalized biological cell-membrane. The article also introduces the construction of mircomotors, including the fabrication methods of materials and their integration with biological tissues (Sun et al., 2020). The control method and movement mode of the micromotors are discussed, and examples of typical micromotors are given, with a focus on their application in the field of biomedicine (Figure 1). The review concludes by proposing some challenges and future prospects for developing a new generation of mirco/nano robtic motors.

## 2 Micro-nano robotic motors based on living cells

Biohybrid robots, which integrate biological entities and synthetic materials, have emerged as a revolutionary development in the field of robotics (Webster-Wood et al., 2022). The power system of organisms in nature has evolved over tens of thousands of years, making it an ideal model for efficient locomotion and adaptation to environmental changes. As a result, the combination of biological tissues and synthetic materials has enabled the creation of motors with these capabilities. The emergence of biohybrid robots has sparked a wave of scientific research, with many scientists achieving remarkable results in their studies (Lv et al., 2023). In the following section, we will discuss the latest advancements in biological components and synthetic materials for micro-nano robotic motors based on living cells.

### 2.1 Microorganism

Micro-nano robotic motors can be categorized into two types based on their driving method. The first type requires external drive which can be limiting due to the need for complex equipment and additional costs (Wang Y et al., 2018; Gao et al., 2021). The second type is known as chemical/catalytic motors, which are powered by intrinsic components such as biological enzymes and redox reactions, generating gas to propel the robot (Tang et al., 2020). As they do not require external equipment, self-propelled micromotors are more convenient for practical applications.

Asymmetric materials have emerged as promising candidates for self-propelled micro-nano robots in recent years. Hahn’s team has developed an asymmetric micro-nano robot powered by urea, which used urea in the stomach as a bioavailable power source to penetrate the gastric mucosal layer (Choi et al., 2021a). However, these materials were often plagued by low biocompatibility and potential biotoxicity of foreign substances. Therefore, the development of biocompatible micro-nano robots is crucial for their widespread use, particularly in overcoming physiological barriers and facilitating efficient drug delivery.

**FIGURE 2:**
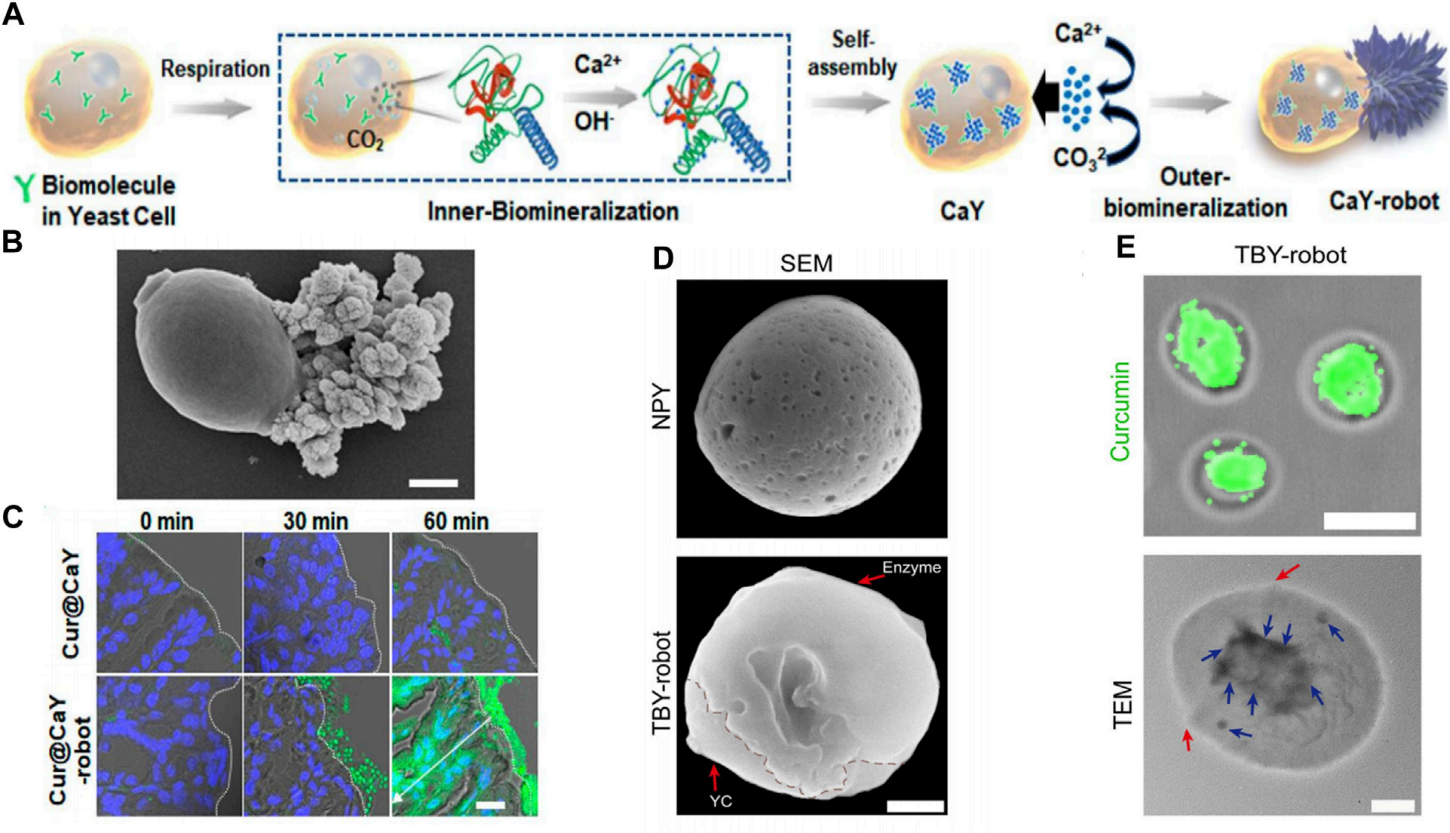
**(A)** Mechanism of yeast-cell-based Cur@CaY-robot. **(B)** SEM images of CaY-robots. **(C)** Histological section of the stomach at different times after the oral intervention. Reproduced with permission. Copyright 2023, ACS nano. **(D)** SEM images of CaY-robots. **(E)** Fluorescence image (top) of TBY-robot loaded with Cur. TEM image (bottom) of a TBY-robot. Red arrows indicate the boundary of conjugated enzymes. Blue arrows indicate the packaged NPs. Reproduced with permission. Copyright 2023, Science Advances.

Yeast cells are single-celled microorganisms that are highly compatible and degradable by living organisms. They are capable of regulating and inducing the biosynthesis of various inorganic nanostructures in cells through a simple and safe synthesis process. Zhang et al. designed a self-propelled yeast cell (CaY) micromotor (Zhang L et al., 2023), (Cur@CaY-robot) (Figure 2A), through dual-biomineralized and acid catalysis of calcium carbonate (CaCO_3_). Under mild conditions, the cellular respiration of nano-CaCO_3_ in yeast cells was biomineralized, providing a nano-scaffold for high-encapsulation curcumin (Cur,a popular anti-inflammatory drug). Simultaneously, the CaCO_3_ crystals outside the yeast cells provided an asymmetric power source for self-propulsion through uniaxial growth.

In the case of micromotor, yeast cells were responsible for loading Cur, while the asymmetric outer layer of CaCO_3_ crystals produced a significant amount of carbon dioxide (Figure 2B). This allowed Cur@CaY-robot to move efficiently in gastric acid and penetrate deeply into the thick gastric mucus, thus improving drug accumulation in the stomach wall tissue and effectively treating gastritis. Additionally, the calcium ions released by the robot synergistically restored gastric motility in mice with gastritis. The yeast micro-nano robot has excellent biocompatibility and biodegradability, as well as good drug-loading ability (Figure 2C).

In addition, micromotors based on microalgae (Choi et al., 2023) and sperm (Xu et al., 2018) have also been widely designed and adopted. Microalgae with multiple flagella have been utilized as a micromotor platform due to their excellent biocompatibility, fast movement without the need for toxic fuel, long lifespan, and versatility in various aqueous environments. As well, some sperm utilize their flexible flagella to generate traveling deformation waves, allowing them to swim in the opposite direction. The sperm cell also possesses a remarkable capability to encapsulate hydrophilic drugs. This unique feature allows the sperm membrane to safeguard drugs from dilution in body fluids, immune reactions, and enzymatic degradation. These abilities and features endow them with multiple functions such as drug delivery, antitumor therapy, and promotion of wound healing.

### 2.2 Macrophage

Living cells have developed unique abilities, such as an endogenous biological motor. Macrophages, for instance, are a cell type with migratory and chemotactic properties that can follow chemokine concentration gradients to target lesion sites and penetrate biological barriers, eliminating the need for hazardous fuels or complex actuation devices (Shi and Pamer, 2011).

Enzymes have been increasingly used as catalytic bioengines due to their capability of converting biocompatible substrate biofuels into driving forces (Hortelao et al., 2021; Arqué et al., 2022; Wang H et al., 2022). These enzyme-driven robots have been successfully created by immobilizing enzymes asymmetrically on the surface of inorganic substances or by asymmetrically polymerizing enzymes into aggregates that display attraction at specific barriers. Additionally, these robots can respond to enzyme-substrate gradients, mimicking the collective driving force exhibited by humans. However, the limitation of single-enzyme bioengine drive restricts self-propelled micro-nano robots to local physiological environments. Adapting to changes in the microenvironment becomes challenging when attempting to reach distant or deep lesions that are separated by multiple biological barriers, such as in gastrointestinal (GI) inflammation including chronic gastritis and inflammatory bowel disease.

Zhang et al. developed a self-propelled and self-adaptive twin-bioengine yeast micro-nano robot (TBY-robot) that could navigate autonomously to sites of gastrointestinal inflammation through enzyme-macrophage switching (EMS) (Zhang W et al., 2023) (Figure 2D). Glucose peroxidase (GOx) and catalase (Cat) were commonly used as feed additives to relieve oxidative stress, maintained microbial system homeostasis, and enhanced intestinal function. The researchers synthesized a TBY-robot by immobilizing GOx and Cat asymmetrically on the surface of anti-inflammatory NP-packed YCs (NPY). The Janus-distributed enzymes could break down glucose to create localized glucose concentrations that could be used to power the TBY-robot. When there was a gradient of glucose concentration in the intestine, the micro-nano robots could pass through the intestinal mucosal barrier and transport across the intestinal epithelial barrier by microfolded cells. Once they arrived at the designated site, the TBY-robot would switch to the macrophage biological engine and autonomously migrate to the inflammatory site of the gastrointestinal tract through a chemokine-mediated macrophage relay.

Notably, the EMS was a crucial component for the TBY-robot to carry out active and targeted anti-inflammatory drug delivery. The small intestine was the only area where a glucose concentration gradient exists due to glucose digestion and absorption, while the colon and stomach lacked a glucose power source. As a result, these robots could not directly access inflammatory sites in the colon and stomach through single-enzyme bioengines. But the TBY-robot with enzyme actuation and macrophage relay can cross multiple biological barriers and autonomously navigate to long-distance and deep lesions. Additionally, the TBY-robot’s enteric coating prevented the inactivation of the enzyme biological engine, stoped the drug from penetrating into the gastric juice which evaluated by Cur loading effciency (Figure 2E), and could not simultaneously release the drug directly in the alkaline colonic environment.

The study found that the micro-nano robots’ lymphatic-blood transport pathway was confirmed through the blockade of microfolded cell transport and splenectomy. In animal models of colitis and gastritis in mice, the TBY-robot showed promising results in reducing inflammation and improving disease pathology. The TBY-robot had features of enzyme activation and macrophage chemotaxis, specifically EMS ability, which allowed it to adapt to changes in the environment, penetrated multiple biological barriers, reached deep inflammatory sites, and provided precise treatment for gastric and intestinal inflammation. This TBY-robot was a safe and versatile delivery tool for the treatment of gastrointestinal inflammation and other inflammatory diseases.

### 2.3 Stem cell

Mesenchymal stem cells (MSCs) have the potential to differentiate into multiple cell types and are often used for cartilage regeneration (Filardo et al., 2016). They can also reduce inflammation and treat damaged cartilage through chondrogenic differentiation and peripheral tolerance induction. This therapy can delay or avoid the need for cartilage replacement surgery. As the number of cells in the lesion is one of the critical parameters, researchers found that the low targeting efficiency of MSCs requires invasive procedures and the use of large numbers of cells combined with intra-articular injection or stent implantation (Goldberg et al., 2017).

To improve the targeting of MSC therapy, researchers were using magnetically driven micro-nano robots. These robots helped target MSC delivery, support MSC through porous structures, and used electromagnetic actuation (EMA) system composed of multiple electromagnetic coils to make the targeting of MSCs precise. These micromotors had shown biocompatibility ability to carry cells, and cell transplantation *in vitro* or in simple *in vivo* environments (Li et al., 2018b).

**FIGURE 3:**
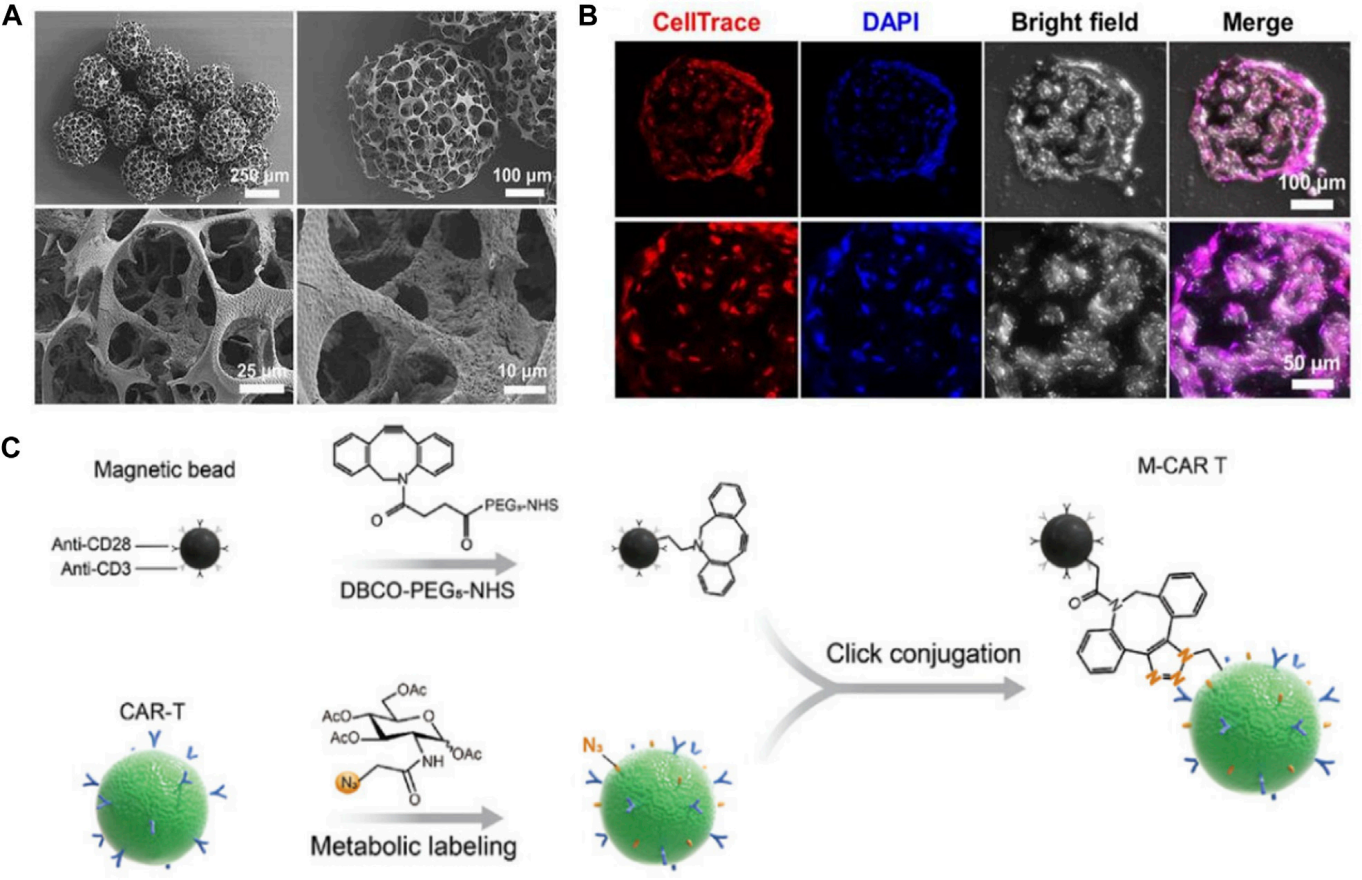
**(A)** SEM images of magnetic microrobots. **(B)** Confocal images of MSCs that were incubated in microrobots for 24 h. Reproduced with permission. Copyright 2020, Science Robotics. **(C)** Schematic illustration of M-CAR Ts’structure. Reproduced with permission. Copyright 2023, Advanved Materials.

In their study, Go G et al. developed a micro-nano robot system for knee articular cartilage regeneration using human fat-derived MSCs (Go et al., 2020). The effectiveness of the system was verified through *in vivo* experiments, with consideration given to its feasibility for clinical trials. The micro-nano robotic system allowed for targeted delivery of MSCs via needle-based injection and magnetic targeting. The system included a magnetically driven micro-nanorobot for cell loading, an EMA system for targeted MSC delivery in accordance with clinical treatment procedures. The micro-nano robots were created by adsorbing magnetic micro-clusters on the surface of PLGA micro-stents (Figure 3A). The microrobot’s porous microstructure could support MSCs (Figure 3B)and inject them into the joint cavity. It also had magnetic driving capabilities provided by ferrous ferulate and positively charged chitosan adsorbed on its surface, forming magnetic micro-clusters. Ferumoxytol, an FDA-approved superparamagnetic oxidized nano-iron drug, was coated with polydextrose sorbitol carboxymethylether and magnetic nanoparticles (MNPs). And the chitosan, a natural multifunctional polysaccharide and representative cationic polymer, was biocompatible and degradable. The magnetic microclusters had adsorbed micro-nanorobots with stronger magnetic properties compared to previous studies. These micro-nanorobots ensured activity, degradability, proliferation, and chondrogenic differentiation of MSCs. In order to improve the effectiveness of stem cell-based cell transplantation, a microrobot-mediated MSC delivery system was developed and tested in an *in vivo* model using small mammalian knee articular cartilage. The system included a targeting mechanism and a magnet designed to immobilize the microrobot at the site of the cartilage defect after positioning. The size and position of the magnet were optimized based on the location of the defect, and the magnetic strength was tailored to the microrobot. Results showed that the combination of microrobots and targeting devices led to higher targeting efficiency of stem cells and improved therapeutic capability.

### 2.4 Other cells

Chimeric antigen receptor T (CAR-T) cell therapy has demonstrated promising results in treating hematological malignancies. However, its efficacy in treating solid tumors has been limited by the immunosuppressive microenvironment and physical barriers. CAR-T cells must navigate a complex vascular system in high-speed blood flow to reach the tumor tissue, which significantly reduces their delivery efficiency (Turley et al., 2015; Ma et al., 2019; Larson and Maus, 2021). Therefore, an ideal CAR-T therapy requires a new type of cell that can successfully navigate the circulatory system, penetrate the tumor tissue, and survive in the harsh tumor environment to exert sufficient antitumor effects.

Recent studies have shown that magnetic particles modified with T cell activators anti-CD3 and anti-CD28 can effectively induce antigen-specific expansion of T cells in tumor tissue through magnetic guidance and actuation (Kalamasz et al., 2004). These modified magnetic particles also provided the bioactive micro-nanorobot with an asymmetric structure and greater acoustic impedance mismatch with medium, making it suitable for ultrasound-driven controllable motion (Wu et al., 2014). This magnetic-acoustic power can be used to manipulate CAR-T cells and facilitate spatial targeting and penetration of solid tumors. In order to enhance the efficacy of CAR-T cell immunotherapy in solid tumors, researchers have developed a microrobot that utilizes immunomagnetic beads coated with anti-CD3 and CD28 antibodies to promote the proliferation and activation of CAR-T cells in the tumor microenvironment.

Tang et al. developed immunomagnetic bead-engineered CAR-T microrobots (M-CAR Ts) that guided by magnetic-acoustic sequential actuation which exhibited precise anti-flow motion and obstacle avoidance capacity, allowing it to maintain its planned route for targeted delivery (Tang et al., 2023) (Figure 3C). M-CAR Ts therapy had unique acoustic manipulation properties and could penetrate artificial tumor tissue through magneto-acoustic sequences. *In vivo*, M-CAR Ts could be targeted and concentrated in the peritumoral area through programmed magnetic guidance. This allowed for precise propulsion of M-CAR Ts into deep tumor tissue, overcoming the limitation of a single driving method. Overall, M-CAR Ts were versatile platform for precise tumor targeting and tissue penetration, which could enhance cell-based solid tumor therapies.

**FIGURE 4:**
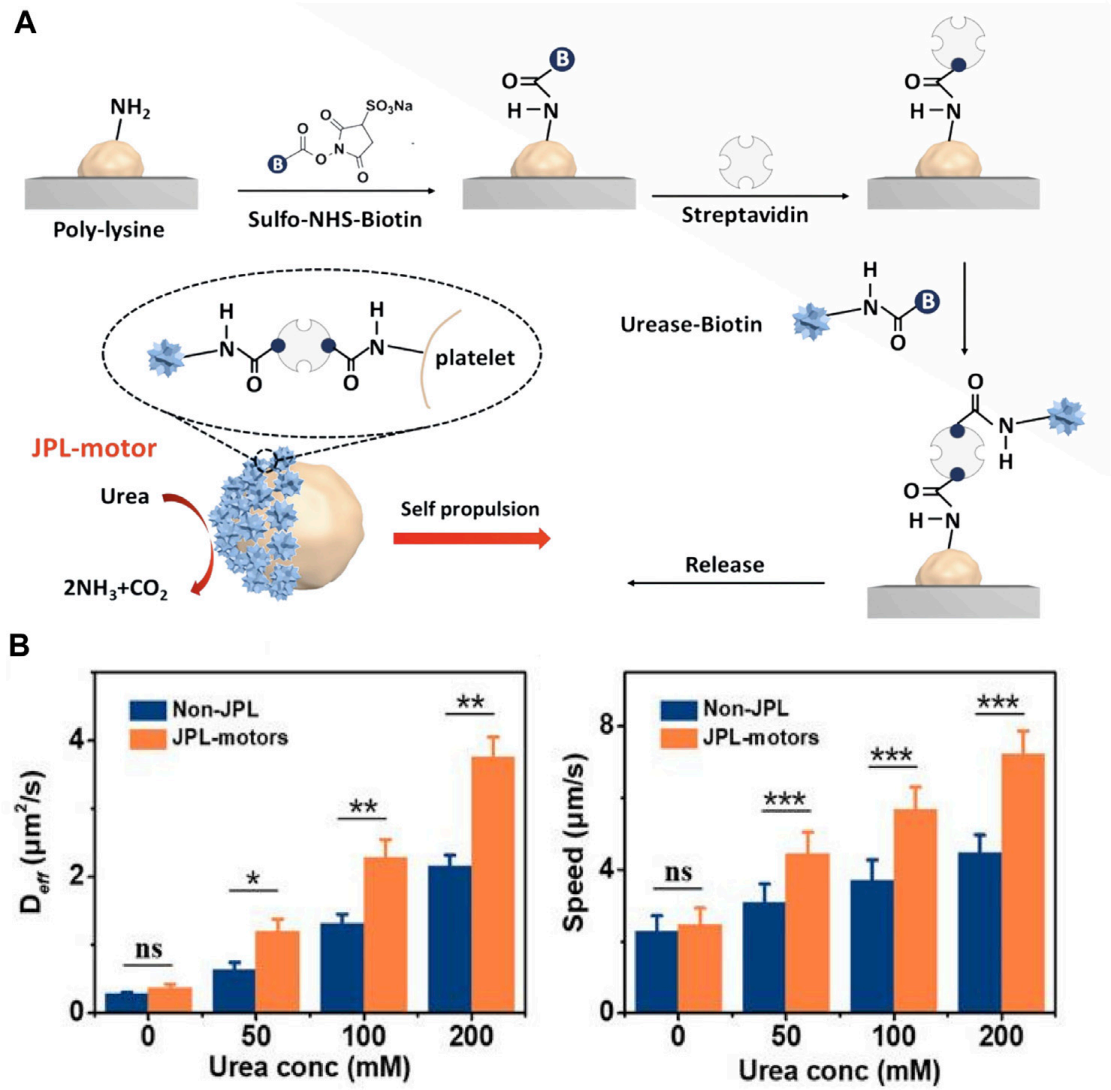
**(A)** Schematic of the fabrication of JPL-motors. Platelets were attached first to a PLL surface and then modified with urease via a biotin-streptavidin-biotin binding complex. **(B)** D_*eff*_ and speed of JPL-motors and non-JPLs in the presence of different urea concentrations. Reproduced with permission. Copyright 2020, Science robotics.

Besides, Tang et al. developed urease-powered Janus platelet micromotors (JPL-motors) (Tang et al., 2020) (Figure 4A). These eliminated the need for external motor, making the robot autonomous and long-lasting. The integration of endogenous enzymes with platelet cells resulted in a biocompatible cell robotic system with unique synergistic capabilities for various biomedical applications. The availability of urea as a common biological substrate in humans, particularly in the urinary system, made this type of microenvironment an attractive option for initial clinical studies. The method proposed in this study offered a straightforward and reliable approach to asymmetrically modify cell surfaces using urease. The Janus structure, which allowed for asymmetric driving forces and active locomotion, was more desirable than full enzymes covering the entire cell membrane.

The current technique for Janus cell modification involved attaching platelets to a poly (L-lysine) (PLL) - modified substrate to block the attached platelet surface and then used biotin-streptavidin-biotin conjugation to modify the exposed cell membrane with urease. This study showed that the use of urea fuel in biocatalytic reactions of surface-bound urease could generate efficient propulsion through chemielectrophoresis. As a result, JPL-motors exhibited efficient locomotion in various biological fluids with the presence of urea, and significantly enhanced propulsion compared to urease-fully modified (non-JPL) platelets due to their asymmetric structure (Figure 4B). The research also indicated that the modification of urease had minimal effects on the functional proteins present on the surface of platelets, which allowed for the design of targeting platforms based on the intrinsic and specific binding of platelets to biological targets. The study demonstrated that JPL-motors fueled with urea could effectively bind to cancer cells and bacteria, leading to an enhanced therapeutic effect when loaded with anticancer and antibiotic drugs respectively.

By combining platelets with a urease biocatalytic engine, a biocompatible and biodegradable cellular robot with self-propelling ability was created. The surface engineering technique of Janus urease coatings was found to be a simple and robust approach to functionalize native cell surfaces asymmetrically, which had a great potential for the development of advanced cellular motors.

## 3 Micro-nano robotic motors based on functionalized biological cell membranes

Since the development of cell membrane coating technology, several methods have been employed to functionalize cell membranes in a non-destructive manner. These methods include lipid insertion, membrane hybridization, metabolic engineering and gene modification, which aim to incorporate diverse functions into natural cell membranes. The modification of membranes is anticipated to augment the multifunctional and multitasking capabilities of cell membrane-coated micromotors, enabling them to better adapt to complex biological environments. The process of preparing functionalized micromotors with cell membrane coating typically involves three main steps: first, the synthesis of active micromotors; second, the preparation of cell membrane vesicles; third, the fusion of the cell nanovesicles and micromotors. This section will address the various designs of cell membrane coatings based on different micromotor designs.

### 3.1 Macrophage membrane

The role of immune cells in controlling tumor progression is gaining recognition, particularly macrophages due to their long half-life and tumor targeting abilities. Depending on the microenvironment, macrophages can differentiate into M1 and M2 phenotypes with distinct functions. M1 macrophages inhibit cancer cell growth while M2 macrophages promote it (Xia et al., 2020; Yang et al., 2022).

**FIGURE 5:**
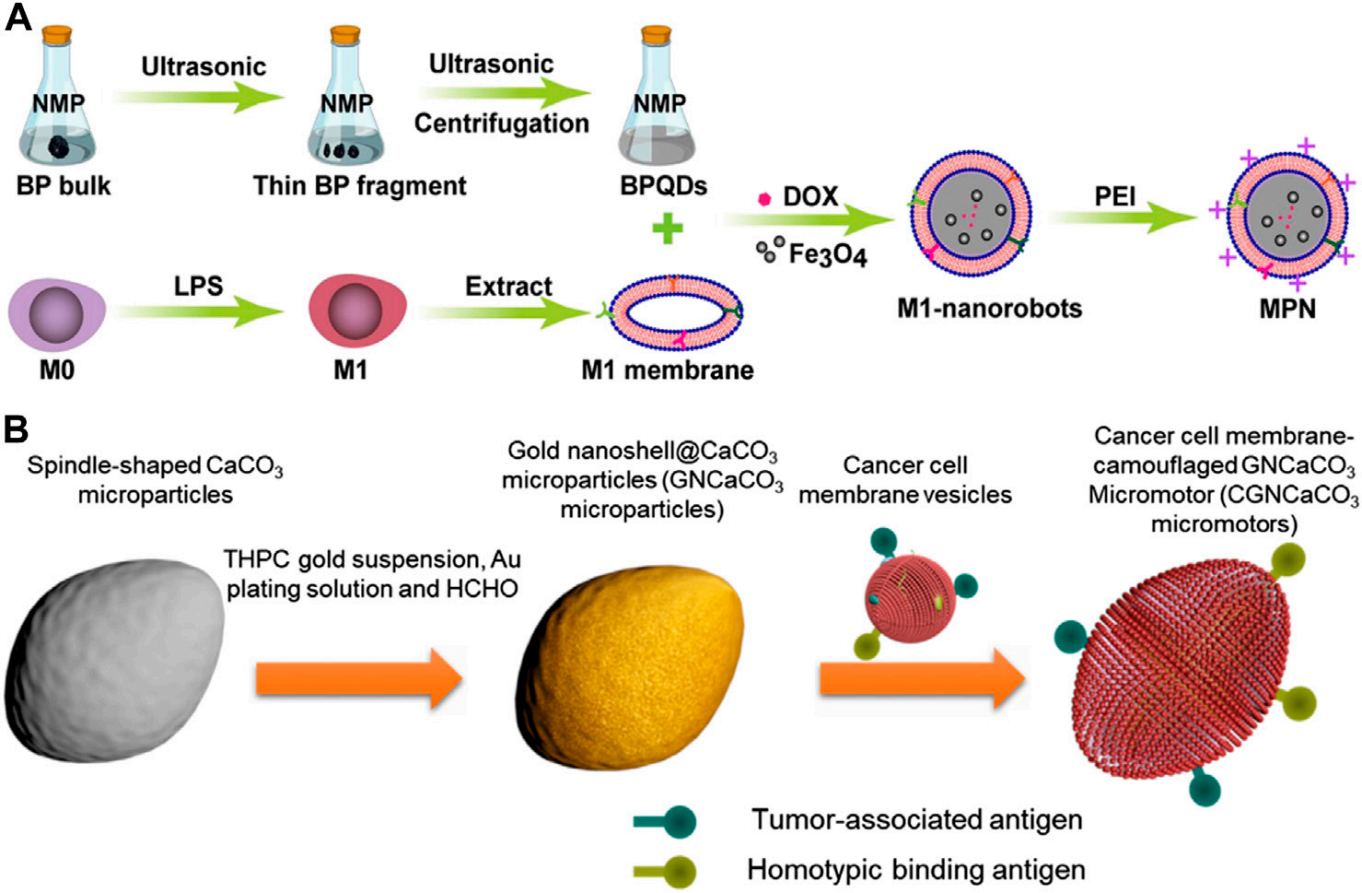
**(A)** Schematic of biomimetic M1 macrophage membrane-camouflaged magnetic nanorobots for cancer therapy. Reproduced with permission. Copyright 2022, ACS Applied Materials&Interfaces. **(B)** Schematic fabrication of acoustic-propelled micromotor based on cancer cell membrane-coated gold nanoshell@CaCO_3_ microparticle. Reproduced with permission. Copyright 2020, Advanced Therapeutics.

Song et al. drew inspiration from M1 macrophages and designed an anticancer micromotor that encapsulated black phosphorus quantum dots (BPQDS) to obtain photothermal and photoacoustic properties, as well as magnetically oxidized nanoparticles for magnetic control (Song et al., 2022) (Figure 5A). The study demonstrated the potential of modified magnetic nanoparticles (MPNs) for effective tumor therapy. The MPNs were modified with positively charged polyethyleneimine (PEI) to facilitate cell phagocytosis. These nanoparticles had good biocompatibility, precise motion control, and excellent photoacoustic (PA) imaging capabilities. Additionally, they could utilize the intrinsic properties of macrophages and the immune properties of M1 macrophages. Under near-infrared radiation (NIR), MPNs could generate high levels of reactive oxygen species (ROS) to exert anti-tumor effects. In a mouse mammary tumor model, antitumor drug doxorubicin (DOX)-loaded MPNs demonstrated a good tumor growth inhibitory effect under external magnetic field and near-infrared irradiation. Overall, the study suggested that MPNs were promising nanotherapeutic platforms for micro-nanorobot tumor therapy.

### 3.2 Cancer cell membrane

Cancer cell membranes coating technology has advantages in cancer drug targeting and imaging due to the homogeneous targeting capabilities provided by membrane antigens (Fang et al., 2018). These membrane antigens also aid in regulating anticancer immunity (Kroll et al., 2017). Additionally, cancer cells are easily cultured and their cell membranes can be easily extracted, further enhancing the benefits of this technology (Fang et al., 2014).

Polymeric nanoparticles have been functionalized with cancer cell membranes to replicate the antigenic composition of the original cancer cells. This design has opened up possibilities for two types of anticancer therapies. Firstly, cancer cell membrane-coated nanoparticles (CCNPs) have been demonstrated to successfully bind tumor-associated antigens to immune adjuvants of antigen-presenting cells (Oroojalian et al., 2021), thereby stimulating anti-cancer immune responses. Secondly, CCNPs contain cell adhesion molecules sourced from cancer cells, enabling them to participate in homotypic binding, a phenomenon observed in cancer cells that causes them to stick together and resulting in tumor growth. The homotypic binding property of CCNPs allows it to be involved in the cell-specific targeting of cancer cells, which is a crucial aspect of localized and directed cancer therapy (Zeng et al., 2022).

H et al. developed micromotors called CGNCaCO_3_, which were made of G422 murine cancer cell membrane-camouflaged gold nanoshell-covered CaCO_3_ (H et al., 2019) (Figure 5B). The fabrication process involved synthesizing CaCO_3_ particles, functionalizing gold nanoshells, and fusing cancer cell membrane vesicles with the GNCaCO_3_ particles. The CGNCaCO_3_ micromotor was able to autonomously move under the exposure of an external acoustic field and accumulate on cancer cells due to its homotypic binding to cancer cell membranes. When injected subcutaneously, the micromotor enhanced the immune activity by camouflaging cancer cells. The CGNCaCO_3_ micromotor possessed biological characteristics similar to those of natural cancer cells, enabling it to promote a diverse range of antigens and biological processes that were not achievable with common synthetic motors.

### 3.3 Red blood cell membrane

Red blood cell (RBC) membranes have been extensively studied as a coating material in various applications such as drug delivery (Aryal et al., 2013), imaging (Rao et al., 2017), photothermal therapy (Piao et al., 2014), and immune modulation (Hu et al., 2013). The RBC membrane coating mimics host RBC and provides superior immune concealment (Chen et al., 2019).

**FIGURE 6:**
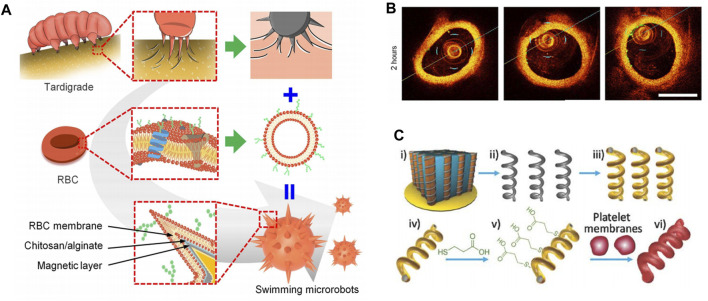
**(A)** Biomimetic design of swimming microrobot. Reproduced with permission. **(B)** Time-lapse IVOCT images of microrobots. Copyright 2023, Science Advances. **(C)** Schematic preparation of magnetic-driven micromotor consisting of platelet membrane-functionalized helical Ni/Au/Pd nanostructure. Reproduced with permission. Copyright 2018, Advanved Materials.

Swimming micro-nano robots in the circulatory system hold great promise in precision medicine. However, they currently face challenges such as limited adhesion to blood vessels, strong blood flow, and clearance by the immune system. These factors reduce the effectiveness of targeted interactions. Li et al. discussed the design of a swimming micro-nano robot inspired by the claw structure of tardigrades (Li et al., 2023) (Figure 6A). The robot featured a claw-engaged geometry, a surface camouflaged with RBC membrane, and magnetically actuated retention with a rotating magnetic field (RMF) for better navigation. These minimized the effects from blood flow. The researchers used intravascular optical coherence tomography (IVOCT) to monitor the activity and dynamics of the microrobots in the jugular vein of rabbits. The results showed that magnetic propulsion was highly efficient even at flow rates of approximately 2.1 cm/s, which was compared with the blood flow characteristics in rabbits. The microrobots remained in the blood vessels for more than 36 h (Figure 6B).

The effect of RBC membrane coating on vascular movement of swimming microrobots was also investigated. In static plasma, the swimming microrobots without RBC membrane coating showed a gentle wobble behavior and remained immobile under the RMF in the *xz* plane (maximum magnetic field of 30 mT). This reflected that the adhesion of the claw-engaged structures in blood vessels was too high to overcome the magnetic movement of the swimming microrobots. In contrast, some swimming microrobots coated with RBC membranes exhibited linear motion in blood vessels, suggesting that the RBC membrane coating effectively regulated the adhesion of swimming microrobots in blood vessels. In conclusion, the controllable motion and magnetic drive retention in blood vessels significantly improved the efficiency of targeted drug delivery.

In addition, RBC membranes have been modified onto various substrates, including magnesium microparticles (Wei et al., 2019), magnetic hemoglobin (Gao et al., 2019), perfluorocarbon nanoemulisions (Zhang et al., 2019) and chitosan-heparin layer-by-layer assembled capsules (Shao et al., 2018). These modifications have resulted in a diverse range of applications, including rapid detoxification, photodynamic therapy, oxygen delivery, and thrombus ablation with multiple functions.

### 3.4 Other cell membrane

Platelets (PL) are known to display unique surface moieties that help them adhere to various disease-associated substrates, which can be linked to vascular injury, immune evasion, and pathogen interactions (Wang et al., 2020). Additionally, platelets play an important role in regulating immune responses, maintaining hemostasis, and participating in wound healing, making them critical players in interacting with many types of cells (Chen and López, 2005; Olsson et al., 2005). Due to their wide range of biological interface capabilities, platelet membrane micromotors have been developed using their membranes. Early studies of PL membrane-encapsulated nanoparticles have shown that they have several essential functions, including reduced cellular uptake by immune cells (Kunde and Wairkar, 2021), subendothelial adhesion (Hu CM et al., 2015), improved tumor targeting (Hu Q et al., 2015), and enhanced affinity for pathogens (Zhuang et al., 2020).

In a recent study, Li et al. fused platelet membrane-derived vesicles (PL-vesicles) onto the surface of helical nanomotors to create PL-nanomotors (Li et al., 2018c). The helical structures were created through Pd/Cu co-electrodeposition in 400 nm pores of polycarbonate films, followed by dissolving the copper, releasing Pd nanostructures, and depositing Ni/Au bilayers to give them magnetic properties (Figure 6C). The synthesized magnetic helical nanostructures were then modified with 3-mercaptopropionic acid (MPA) to provide a negative charge to the Au surface, allowing for effective coating of the helical motor after incubation under ultrasound.

Therefore, its magnetic propulsion was not affected by the presence of biofilms. The study evaluated the propulsion and anti-biofouling abilities of PL nanospheres in whole blood. The results showed that the PL-nanomotor had higher propulsion efficiency compared to the bare nanomotor. The bare nanomotor was hampered by severe biofouling, while the light-emitting nanomotor exhibited efficient magnetic actuation. These findings further supported the protective effect of platelet membrane coating against biofouling in complex biological matrices and suggested potential *in vivo* studies.

## 4 Applications of living cells and functionalized biological cell membranes micro-nano robots

(Table 1) lists applications for various micromotors. When the efficient locomotor capabilities of synthetic micro-nano robots are combined with the biological functions of native living cells or cell membranes, these micromotors gain unique capabilities that greatly enhance their performance in improving various biomedical applications. These applications include drug delivery, immunomodulation, and phototherapy. The following sections will discuss these functions in more detail.

**TABLE 1:** Biofunction and application of mirco-nano robots.

Construction	Biofunciton	Application	References
Sperm	Swim into the tumors	Drug delivery	Xu et al. (2018)
Microalgae	Alleviate the hypoxic condition and modulate the immune responses	Wound healing	Choi et al. (2023)
Macrophage	Phagocytic ability	Cargo delivery	Dai et al. (2022)
Stem cell membranes	Secretion of paracrine factors	Therapeutic cardiac regenertation	Tang et al. (2017)
T cell membranes	Ability to recruit and localize at tumor sites	Enhance drug targeting	Zhang et al. (2017)
Cancer cell membranes	Promote anticancer immune responses	Drug delivery	Fang et al. (2014)
Lung epithelial cell	Surface receptors that pathogens depend on for cellular entry	Neutralize the virus	Zhang et al. (2020)
Leukemia cell	Targeting sites of inflammation	Drug delivery	Park et al. (2021)

### 4.1 Drug delivery

Neutrophils (NE) are crucial in the immune response as they remove infections through various means like phagocytosis, degranulation, reactive oxygen species, and NE extracellular traps (NETs) (Jorch and Kubes, 2017; Papayannopoulos et al., 2018). During inflammation, NE can cross the blood-brain barrier/blood-brain tumor barrier (BBB/BBTB) by following the gradient of inflammatory factors (Deoliveira et al., 2016; Oliveira et al., 2018). NEs have been utilized as drug carriers to target inflammatory tumors due to their chemotactic properties. Although some reduction in tumor size has been observed, complete cure of cancer in mice has not been achieved due to limitations in drug-loading capacity, migration speed, and accumulation efficiency of existing NE carriers (Xue et al., 2017; Chu et al., 2018; Tang et al., 2019). Therefore, NE-based swimming microrobots with significantly enhanced drug loading, locomotor, and navigation capabilities hold promise for active delivery *in vivo*.

**FIGURE 7:**
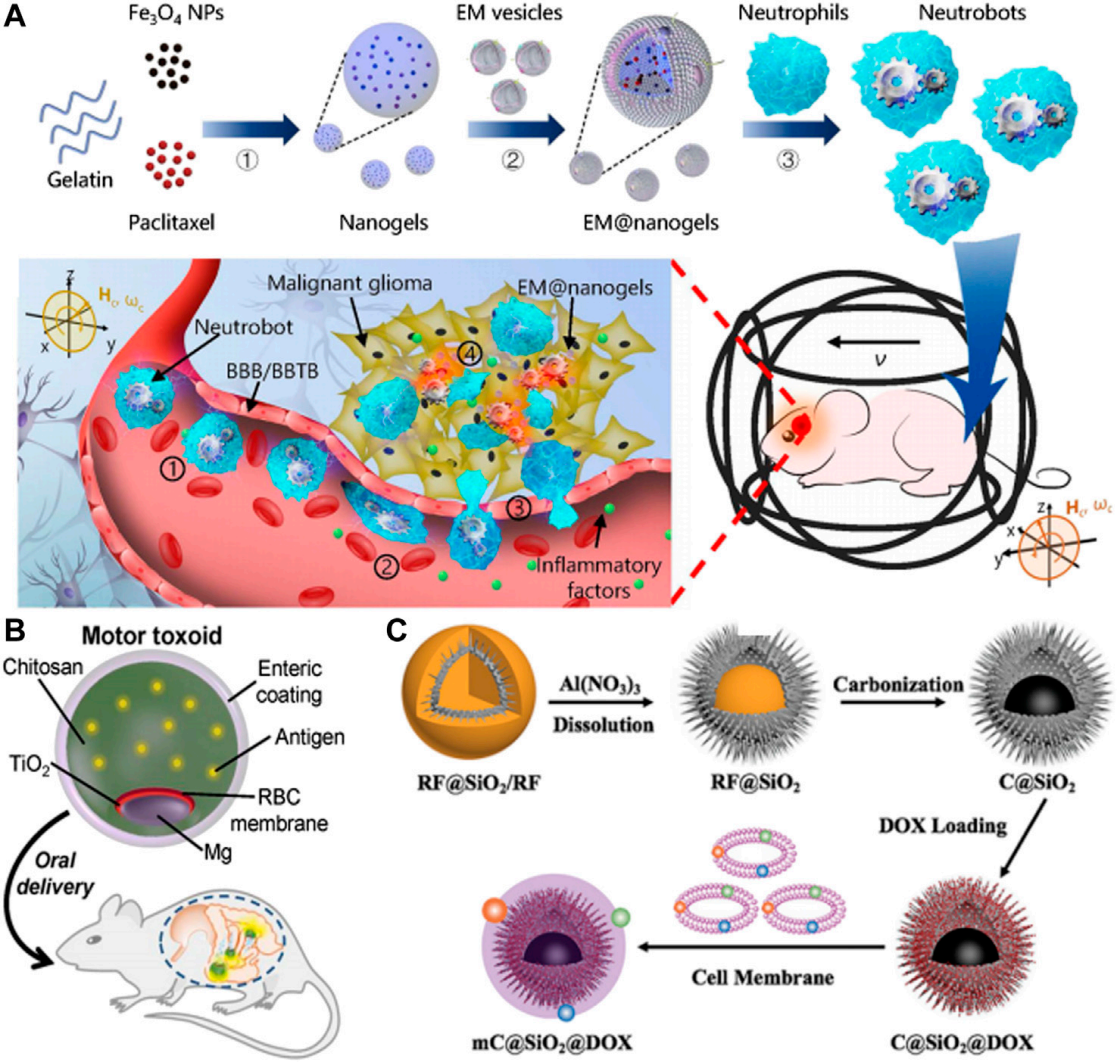
**(A)** Schematic of active therapeutics of dual-responsive neutrobots *in vivo*. Reproduced with permission. Copyright 2021, Science Robotics. **(B)** Toxin binding RBC membrane-coated Mg micromotor (micromotor toxoid) for oral vaccination. Reproduced with permission. Copyright 2019, Nano Letters. **(C)** Schematic illustration of the self-thermophoretic propulsion nanomotors actively seeking and effectively endocytosing into tumor cells under NIR light irradiation, thereby enhancing photothermal and chemotherapy. Reproduced with permission. Copyright 2020, Small.

Zhang et al. developed dual-responsive (DR) hybrid NE microrobots, also were knowed as neutrobots, that could be used for the treatment of active malignant glioma *in vivo* (Zhang et al., 2021) (Figure 7A). These robots had the ability to move intravascularly using magnetic propulsion and exhibit chemotactic behavior along gradients of inflammatory factors. To achieve this, the robots were coated with paclitaxel (PTX) magnetic nanogel, which was coated on the outer membrane of *E. coli (E.coli)* and then phagocytized by NE. The camouflage provided by the *Escherichia coli* membrane prevented the drug from leaking into the NE, resulting in a bioactivity similar to NEs. The neutrobots could be individually propelled and exhibited swarm locomotion in a rotating magnetic field (RMF), and had demonstrated efficient operation in blood *in vitro*. *In vitro* model of the BBB, magnetic actuation was found to significantly increase the accumulation of neutrophils at the site of lesion. Additionally, chemotactic motility allowed for BBB penetration. The efficacy of active targeted delivery was further confirmed *in vivo* through the use of double-reactive neutrophils for the treatment of postoperative glioma. The biohybrid neutrobots developed in this study demonstrated the ability to harness NE’s natural characterisitic, which were otherwise difficult to replicate. Such dual-response neutrobots might improve the efficiency of active targeted delivery and noninvasive precision therapy.

Despited significant progress in intracellular and active targeted drug delivery of micromotors, *in vivo* applications still posed challenges. While RMF had been proven effective for intracellular and targeted delivery of external stimuli, precise directional and temporal control of ultrasound and photostimulation remained difficulties. Additionally, active targeting of chemoattractant micro-nano robots *in vivo* is hindered by the complex coexistence of the circulatory system and various chemical gradients, which makes the chemotactic motility of micro-nano robots unfavorable. To improve practical application, the sensitivity of micro-nano robots *in vivo* must be enhanced for precise motion control with an extended tactical operating range (Choi et al., 2021b).

### 4.2 Immune modulation

The task of creating effective micro-nano robots delivery systems for transporting and enclosing antigenic agents and immunostimulants is a significant challenge in the field of nanomedicine (Fang and Zhang, 2016; Zhou J. et al., 2020). The use of micromotors with enhanced drug loading, sustained release, targeted delivery, and oral inoculation has become increasingly popular.

One effective strategy to immobilize and neutralize toxins to the carrier surface is through cell membrane coating technology. Combining this technology with a micromotor platform has the potential to improve cargo delivery and enhance tissue penetration. A recent development in this field was the unique biomimetic micromotor toxoid strategy for oral vaccines. This method involved preparing micromotor toxoids in a sequential process that confered antigenicity by coating the micromotors with a toxin-inserted erythrocyte membrane.

In Wei et al.s’ study, motor toxoid was administered orally to a mouse and targeted to the gut (Wei et al., 2019) (Figure 7B). Once in the gut, the micromotors were activated and propelled through the surrounding fluid to enhance retention and penetration of the antigen payload. To protect the motor toxoids from activation and degradation in the low pH environment of the stomach, a pH-responsive enteric coating was used. The study evaluated the ability of motor toxoids to induce an immune response to staphylococcal *α*-toxin by administering it along with static microparticle toxoid as a passive control group. Based on the absorbance data of the ELISA assay, it could be inferred that the motor toxoid exhibited a higher production of *α*-toxin antibodies in comparison to the static microparticle toxoid. The IgA titers data indicated that the motor toxoid platform increased IgA production by approximately ten times.

The combination of the cell membrane-based toxin retention platform and micromotor technology showed promise for the development of safe, effective, and easily administered immunotherapeutic agents. This system could extend to safely deliver various toxic antigen cargoes and provides efficient active delivery capability.

### 4.3 Photothermal therapy

In the field of external propulsion, NIR light propulsion has significant potential for biomedical applications. This is due to its ability to penetrate deep into tissues, its biocompatibility, remote control capabilities, ease of operation, and fast response. For instance, Li et al. developed a tubular mesoporous silicon-based micromotor that can be driven by NIR light (Li et al., 2021). These micromotors, when injected into the bloodstream and activated at the site of a lesion using infrared light, gain the ability to move and facilitate their penetration into damaged blood vessels. By utilizing the heat generated from near-infrared light, the micromotor can function as a photothermal ablation agent for inflammatory macrophages.

In addition to optimizing self-propelling and drug loading abilities, micromotors need to possess good antibioadhesion and specific targeting capabilities to serve as effective drug delivery systems. In recent years, cell membrane coating technology has emerged as a promising approach to fulfill these requirements. Zhou et al. had demonstrated a NIR light-driven biomimetic micromotor which was composed of carbon@silica (C@SiO_2_) with semi-yolk@spiky-shell structure loaded with anticancer drug DOX and MCF-7 breast cancer cell membrane (mC@SiO_2_@DOX) for cancer therapy (Zhou M. et al., 2020) (Figure 7C). This mC@SiO_2_@DOX motor was evaluated for photothermal and chemotherapy of breast cancer. Owing to the asymmetric spatial distribution, the biomimetic mC@SiO_2_@DOX nanomotor demonstrated efficient self-generated thermal propulsion capability. Additionally, the coating of MCF-7 cancer cell membrane significantly reduced the bioadhesion of nanomotors in biological media and exhibited highly specific self-recognition to the source cell line. The combination of efficient propulsion and cognate targeting greatly enhanced cell adhesion and increases cellular uptake efficiency from 26.2% to 67.5%. As a result, the biomimetic mC@SiO_2_@DOX nanomotor showed a promising photothermal and chemotherapy synergistic effect, with a growth inhibition rate of MCF-7 cells exceeding 91%. The intelligent design of this fuel-free, NIR light-driven biomimetic nanomotors might open doors for the application of self-propelled nanomotors in biomedicine.

In summary, the homotypic targeting effect of cancer cell membrane-coated micromotors is ideal for localized chemotherapy and phototherapy at tumor sites. Additionally, this targeting capability can also be utilized with other cell membranes, including platelets and leukocytes, for similar therapeutic purposes. The fast motion capability of the micromotor, combined with the specific targeting capabilities of cell membranes, makes it a promising tool for future cancer treatments.

## 5 Future prospects

This article provides a comprehensive overview of the recent advancements in micro-nano robotic motors, covering its fundamental components, control techniques, and initial applications. The field of micro-nano robots is currently undergoing a significant transformation, thanks to the diligent efforts of researchers, which could have far-reaching implications in various domains such as biology, medicine, and engineering. Despite the significant strides made in this field, there are still obstacles that impede laboratory research from translating into real-world applications. For instance, after micro-nano robots complete *in vivo* tasks, their residual presence in the body can be cytotoxic, potentially leading to unintended harmful outcomes. In this case, micro-nano robots made of biodegradable materials have become the trend in healthcare and biomedical applications (Sitti et al., 2015). Therefore, the development of micro-nano robots must consider and tackle several crucial issues to ensure technological progress.

The first problem the limited cell sources available for the biological components of micro-nano robots. Stem cell technology could be a solution, but the high cost of this technology limits its ability to meet market demands. Furthermore, the short lifespan of robots, which cannot exceed several months, affects their practical application value. Developing micro-nano robots that can maintain their locomotion ability for longer periods can address this issue (Halder and Sun, 2019). Therefore, there is a need to draw inspiration from nature to find ways to generate long-term cell cultures and even achieve cell immortalization.

Second, the challenge of maintaining locomotor performance in micro-nano robots is closely tied to the optimization of materials. These materials must meet several requirements, including biocompatibility, stability, durability, and flexibility (Sun et al., 2020). Despite the widespread use of various materials in constructing micromotors, they still struggle to create an environment that effectively induces cell orientation or differentiation, similar to that provided by organisms. In response, material science and fabrication techniques should be integrated more closely to develop more advanced materials with bioinspired form and function.

The third issue in micro-nano robots concerns the limitation of practical applications due to the culture and testing of robots in liquid environments. While attempts have been made to manipulate the atmospheric conditions by encapsulating biological entities in closed media, the resulting biohybrid systems are only viable for a few days (Li et al., 2023). To overcome this limitation, researchers are exploring the development of a human-like built-in circulatory system or biomimetic vascular network. This would enable effective detachment from the culture medium environment. Meanwhile, current micromotors are mainly designed for a single function, whereas the potential of robotics lies in building complex systems that can perform multiple functions to adapt to various environments.

Micro-nano robots have demonstrated impressive performance and hold practical potential across various fields. Future research should aim to enhance their performance and explore their applications in biological and medical domains. This review aims to inspire multidisciplinary researchers to address these issues and foster the development of micro-nano robots. We are confident that micro-nano robots will continue to achieve exciting advancements in the future.
